# Definitive radiotherapy and Single-Agent radiosensitizing Ifosfamide in Patients with localized, irresectable Soft Tissue Sarcoma: A retrospective analysis

**DOI:** 10.1186/1748-717X-5-55

**Published:** 2010-06-16

**Authors:** Franziska Eckert, Christiane Matuschek, Arndt-Christian Mueller, Martin Weinmann, Joerg T Hartmann, Claus Belka, Wilfried Budach

**Affiliations:** 1 Eberhard-Karls-University Tuebingen, Department of Radiooncology, Hoppe-Seyler-Str. 3, 72076 Tuebingen, Germany; 2 Heinrich-Heine-University Duesseldorf, Department of Radiooncology, Moorenstr. 5, 40225 Duesseldorf, Germany; 3 Christian-Albrechts-University, Medical Oncology Center, Comprehensive Cancer Center North, Arnold-Heller-Straße 3, 24105 Kiel, Germany; 4 Ludwig-Maximilians-University Muenchen, Department of Radiooncology, Marchionistr. 15, 81377 Muenchen, Germany

## Abstract

**Background and Purpose:**

Standard therapy for soft-tissue sarcomas remains complete resection. For primary radiotherapy local control rates of 30-45% have been reported. We analyzed retrospectively 11 cases of radiochemotherapy with single-agent ifosfamide in patients with macroscopic soft-tissue sarcomas.

**Patients and Methods:**

The patients were treated in irresectable high risk situations. Radiation therapy was performed with median 60 Gy. During the first and fifth week the concomitant chemotherapy with ifosfamide was added. Two patients received trimodal therapy with additional regional hyperthermia.

**Results:**

The therapy was completed in 73% of the patients. Average local control time was 91 months, median disease-free-survival/overall-survival was 8/26 months. Five-year rates for local control/disease free survival/overall survival were 70%/34%/34%. The limited prognosis is mainly caused by systemic treatment failure.

**Conclusions:**

The data strongly suggest a better outcome of radiochemotherapy with ifosfamide compared to radiotherapy alone and radiotherapy in combination with other radiosensitizers.

## Introduction

Advanced, localized soft tissue sarcomas are still a challenge for all therapeutic disciplines involved. The multimodal therapy consists of surgery, radiotherapy and chemotherapy [1]. Radiotherapy improves local control as neoadjuvant and adjuvant approach in many clinical situations, especially in high grade sarcomas and deep seated tumours.

Adjuvant chemotherapy is applied by some groups in selected patients with sarcomas of the extremities in high risk situations, for example deep location, grade 2 and 3 and size larger than 5 cm [2-4]. Despite all efforts, the prognosis, especially for advanced high grade sarcomas, is still limited. In localized disease the therapeutic aim is to achieve a complete resection as most important prognostic factor for local control and survival. Some authors even state that only complete, margin-negative resection can be considered as curative treatment [5].

The question is how to treat patients with localized, non-resectable tumours. As the disease is not metastasized, a chance for cure can be assumed, but long-term tumour control could only be achieved with radiation doses of at least 63 Gy [6]. This dose-response-relationship reveals the difficulty of treatment, because extent of the tumour and proximity to organs at risk limit the curative approach especially in retroperitoneal sarcomas.

The first report on a series of 36 irresectable patients treated with concurrent radiochemotherapy was published in 1991. Aggressive treatment in large, irresectable soft tissue sarcoma showed to be favorable compared to the application of hypofractionated palliative regimens [7].

Effective chemotherapeutic regimens were initially developed for the use in metastatic disease. Considering different chemotherapeutic agents, our selection was geared to the nowadays established first-line chemotherapeutic approach consisting of anthracyclines and ifosfamide [4,8]. Ifosfamide was chosen as radiosensitizer due to its superior toxicity profile in combination with radiotherapy [9,10]. This treatment combination was applied in individual cases without alternative options with the informed consent of the patients, that it is no standard regimen.

The aim of our analysis was to evaluate retrospectively the safety and efficacy of simultaneous radiochemotherapy with single-agent ifosfamide. To our knowledge, this is the first approach of definitive ifosfamide-based radiochemotherapy in patients with localized, irresectable soft tissue sarcomas.

## Patients and Methods

From 1996 to 2007 eleven patients (four males, seven females), median age of 55 years, were treated with concurrent ifosfamide and radiotherapy as definitive treatment primarily or after resection with gross residual tumour. Median follow-up was 55 months ranging from 4 to 131 months.

The tumour characteristics defined a high risk situation regarding all relevant prognostic parameters. All patients had sarcomas larger than 5 cm. The tumours were graded G2 or G3 according to FNCLCC (Fédération Nationale des Centres de Lutte Contre le Cancer) (73% G3). Five tumours had maximal initial diameters of more than 10 cm, one of which was partially resected before radiotherapy resulting in a residual tumour of 4 cm. Thus, four tumours (36%) were larger than 10 cm before radiotherapy. Details are summarized in table 1.

**Table 1:** Patients' characteristics

Age *(years)*		
Median	55	
Range	36-64	
**Gender**		
Male	4	36%
Female	7	64%

**T-category**		
T1	0	0%
T2	11	100%

**Initial tumor size**		
5 - 10 cm	6	54%
>10 cm	5	46%

**Grade acc. FNCLCC**		
Grade 1	0	0%
Grade 2	3	27%
Grade 3	8	73%

**Localisation**		
Craniocervical	2	18%
Trunk	9	82%

**Follow-up *(months)***		
Median	55	
Range	4-131	

The diagnostic work-up included cross-sectional imaging of the tumour region and chest X-ray or computed tomography to exclude pulmonary metastasis. The tumours were diagnosed histologically. The treatment options were coordinated with all disciplines involved and surgical options were excluded. Routinely WHO (World Health Organization) performance status and laboratory parameters including creatinine clearance were assessed to ensure sufficient organ function for chemotherapy.

Radiotherapy was planned 2-D (Two-dimensional) or 3-D (Three-dimensional)-conformal. In two cases better sparing of normal tissue in the head and neck region and the retroperitoneum was achieved by IMRT (Intensity Modulated Radiotherapy). Reproducible immobilization and linear accelerators with 6-15 MV (Mega Voltage) photons were used in all patients. Median radiation dose was 60 Gy (range 50.0-72.6). Fractionation schedules were 1.8 or 2.0 Gy/day, five times a week. Two patients were treated with hyperfractionated therapy twice-daily.

Total radiation dose was aimed to be 60 Gy or higher, dose adaptations were made to achieve sparing of organs at risk. The aim was a safety margin of 2 cm in all directions. It was reduced in case of respected anatomical borders or for sparing of dose-limiting organs at risk. Accepted dose for spinal cord was 45 Gy, for small bowel 45-50 Gy and 12 Gy for at least one kidney.

**Figure 1:**
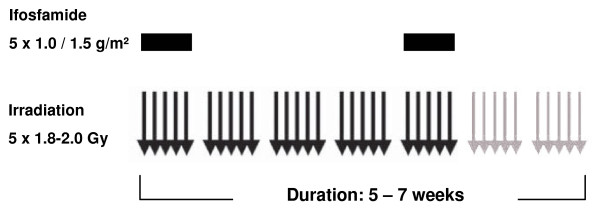
***Treatment Flow Chart***. Treatment consisted of five to seven weeks of radiotherapy (median total dose 60 Gy) with two courses of chemotherapy in the first and fifth week of irradiation. One chemotherapy course consisted of five applications of 1.0 or 1.5 g/m^2 ^Ifosfamide at five subsequent days. For two patients locoregional hyperthermia was added.

The cumulative ifosfamide dose of 10 or 15 g/m^2 ^was administered in two different schedules with 1.0 or 1.5 g/m^2 ^on five subsequent days in the first and the fifth week. Two patients received concurrent regional hyperthermia. Two hyperthermia treatments weekly were prescribed. In one patient temperature was measured invasively, in one patient with pelvic manifestation the probe was placed in rectum, bladder and vagina. One patient discontinued hyperthermia after the first treatment due to cardiovascular problems. One received 5 treatments of hyperthermia (25-40 min), the application was limited by severe pain and circulation problems. (Table 2, Figure 1).

**Table 2:** Therapy modalities

Irradiation dose *(Gy)*		
Median	60	
Range	50.0-72.6	
**Irradiation technique**		
3D conformal	9	82%
IMRT	2	18%

**Ifosfamide dose (intended) *(g/m^2^)***		
10	5	45%
15	6	55%

**Additional therapy modalities**		
Hyperthermia	2	18%

Statistical evaluation was performed with SPSS 15.0 to calculate Kaplan-Meier-plots. Local control, disease free survival and overall survival were calculated from the first day of treatment. The comparison of subgroups was done with Log-rank (Mantle-cox) test.

## Results

**Figure 2:**
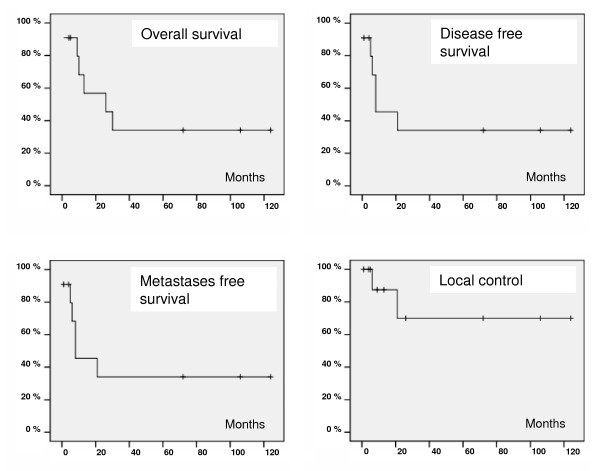
***Survival Data***. Overall survival (a), disease free survival (b), metastases free survival (c) and local control rate (d) of all analyzed patients are shown as Kaplan-Meier-estimation. Estimated 5-year-overall-survival was 34%, disease free survival and metastases free survival 34%, local control rate 70%.

Median follow up of patients alive was 13 months. Estimated local control rate after 2 and 5 years was 70%. Four patients were still at risk after 2 years. A plateau was reached after 22 months. Two of eleven patients had local relapse in the irradiated area after a time of 6 and 21 months, respectively, after radiation doses of 70.2 Gy and 66 Gy. Six patients (54%) died of metastasized disease. Estimated disease free survival was 34% at a plateau after 20 months. Median disease free survival was 8 months as was median metastasis free survival. Median overall survival was 26 months, the estimated five year-overall survival was 34%. No deaths occurred later than 38 months after treatment. At the time of analysis 3 of 11 patients are still alive and disease free, all of them for more than 5 years (Figure 2).

Radiotherapy could be applied as planned in 9/11 (82%) of patients. One patient died during therapy due to respiratory failure caused by enlarging tumour of over 20 cm in the thoracic irradiation field, compressing the lung and the great vessels. In one case radiotherapy could only be completed after a major delay of 4 weeks because of local infectious complications at the tumour site after multiple surgical procedures and osteosynthesis with revisions. The second cycle of ifosfamide was withheld in both patients and due to leucopenia CTC (Common Toxicity Criteria) grade III in one additional patient. Thus, the complete regimen was given in 8/11 (73%) of the patients. Evaluation of skin toxicity was possible for 9 of the patients, 2 of which had severe reactions CTC grade III/IV.

Because of the small sample size, a meaningful subgroup analysis was not possible. However, a trend towards decreased disease free survival in high grade tumours was observed (p = 0.37) (table 3).

**Table 3:** Individual treatment results

Histology	Localisation	Stage/Grade	Maximal diameter Initial - before RT	Irradiation dose	Total ifo dose	Toxicity ≥ °III	Local control *(Mo)*	Overall survival *(Mo)*
***Primary RTCHX***								

Leiomyos.	HN	T2 G3	8 cm	70.0 Gy	7.5 g/m^2^	Skin toxicity °III	72	72 AWOD
PNET	Trunk	T2 G3	11 cm	60.0 Gy	15 g/m^2^		9	9 DOD
Anaplastic S.	Trunk	T2 G3	9 cm	60.0 Gy HF + HT	10 g/m^2^		124	124 AWOD
Angios.	Trunk	T2 G2	8 cm	55.6 Gy	5 g/m^2^	Interruption (abscess)	106	106 AWOD
Chondros.	Trunk	T2 G2	13 cm	66.0 Gy	10 g/m^2^	Leucopenia	21 LF	30 DOD
Synovials.	Trunk	T2 G2	25 cm	50.0 Gy	7.5 g/m^2^	Death during therapy	1	1 DOD
Pleomorphic S.	Trunk	T2 G3	11 cm	70.2 Gy	10 g/m^2^	Skin toxicity °III	6 LF	10 DOD

***RTCHX after R2-resection***								

Leiomyos.	Trunk	T2 G3	15 cm - 4 cm	66.0 Gy HF + HT	10 g/m^2^		26	26 DOD
Synovials.	Trunk	T2 G3	9 cm - 3 cm	50.4 Gy	15 g/m^2^		13	13 DOD
Rhabdomyos.	HN	T2 G3	6 cm - 4 cm	72.6 Gy	15 g/m^2^	Leuco- penia °IV	5	5 DOD
Leiomyos.	Trunk	T2 G3	11 cm	59.6 Gy	15 g/m^2^		4	4 DOD

Abbreviations:

AWOD - Alive without disease

DOD - Dead of disease

HF - Hyperfractionation

HN - Head and neck

HT - Hyperthermia

Ifo - Ifosfamide

LF - Local failure

Mo - Months

PNET - Peripheral neuroectodermal tumor

RT - Radiotherapy

RTCHX - Radiochemotherapy

The two patients experiencing local failure had tumours with diameters over 10 cm. The patients still at risk after 2 years all had tumours smaller than 10 cm. Thus, a trend towards better outcome for the patients with smaller tumours can be assumed.

**Figure 3:**
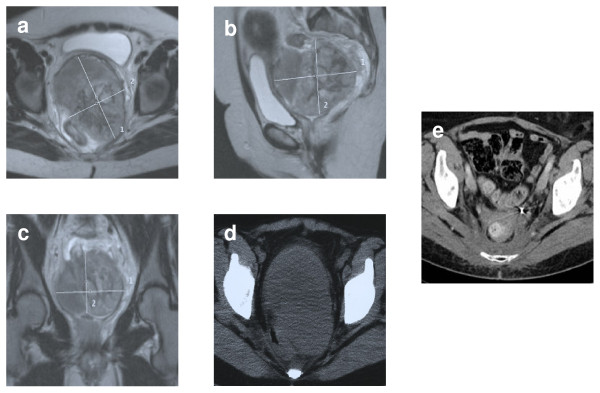
***Case Report***. A 49-year-old female presented with obstipation, vaginal bleeding, urinary problems and chronic vaginal fluor. Assuming a gynaecological tumour a biopsy of the cervix was performed and showed a high grade pleomorphic sarcoma, NOS. The MRI revealed a 13 × 9 × 9 cm tumour between cervix, bladder and rectum (a-d). Due to the palliative intent a debulking of about 80% of the tumour was performed. Prior to radiooncological treatment a protective colostomy was performed. Radiochemotherapy to 60 Gy (hyperfractionated twice daily) was well tolerated, hyperthermia had to be discontinued after the first application due to circulation problems. The colostomy could be removed after therapy. The only relevant late effect of CTC grade III or higher was a unilateral ureteral stenosis treated with a Double-J-catheter on the respective side. Ten years after the therapy the patient was still free of disease (e).

Figure 3 shows MRI (Magnetic Resonance Image)- and CT (Computed Tomography)- scans of a 49-old female patient treated with a radiation dose of 60 Gy with a hyperfractionated twice-daily regimen in combination with ifosfamide and regional hyperthermia after partial resection of a retroperitoneal pleomorphic high grade sarcoma (stage pT2b N0 M0) at the time of diagnosis and eight years after therapy. Hyperthermia was planned twice a week, but had to be discontinued due to circulation problems after the first treatment. Additional chemotherapy with three courses of ifosfamide and epirubicin was administered after completion of radiochemotherapy. At the time of analysis the patient was alive and well without tumour recurrence. The only relevant late effect of CTC grade III or higher was a unilateral ureteral stenosis treated with a Double-J-catheter on the respective side.

## Discussion

Radiochemotherapy with single-agent ifosfamide is a feasible treatment scheme for inoperable high-risk patients with soft tissue sarcomas located in the retroperitoneum or the head and neck region.

The patients belonged to a high risk group associated with an unfavorable prognosis due to the presence of negative prognosticators in soft tissue sarcomas with respect to location, size, depth of infiltration and grading [11]. Low grade tumours were not included. The most negative factor was irresectability respectively incomplete resection. This fact also explains the number of early death observed in 4 patients.

The use of radiosensitizing agents in patients with soft tissue sarcomas can be tracked to the late 80 ies, when for the first time aggressive treatment schedules for irresectable soft-tissue sarcomas were investigated [12]. Goffman et al. described a better outcome and less toxicity for iododeoxyuridine compared to misonidazole. However, local control after 3 years was 37%, thus worse than the here described survival rates [7]. Also the use of razoxane or continuous administration of doxorubicin was discussed in the 90 ies [13,14]. Ifosfamide yielded an at least additional effect in combination with fractionated radiotherapy in a xenograft model [15]. It was used in combined treatment regimens with multi-drug-therapy plus irradiation since 1999 [16,17]

Radiotherapy alone yielded no local control after 5 years in retroperitoneal sarcomas [18]. In head and neck sarcomas, individual cases (1/6 patients) demonstrated a curative potential of radiotherapy alone [19].

Kepka et al. described radiotherapy without additional systemic therapy in 112 patients, 33 of whom had tumours of 10 cm or more before radiotherapy (29%). 11% of the tumours were low-grade sarcomas, the rate of G3 tumours was 37%. The local control rates were 45% and 10% for tumours with diameters from 5 to 10 cm and more than 10 cm respectively after 5 years [20]. Due to the small patient numbers no separate evaluation could be performed with our data, but the estimated overall local control rate of 70% after 5 years in a patient group with 36% of tumours greater than 10 cm and 73% high grade sarcomas gives at least indirect evidence that ifosfamide improves local control in combination with radiation therapy.

A five-year overall survival of 34% of the patients in this prognostic group thus compares favorably with historic controls of radiotherapy alone. The prognosis is determined by systemic treatment failure as 66% of the patients developed distant metastases, explaining why local control only partly translates to overall disease control. According to the latest meta-analysis concerning adjuvant chemotherapy in resected soft tissue sarcoma, an additional doxorubicin and ifosfamide-based chemotherapy regimen significantly reduced distant metastases and mortality in resected sarcomas [21,22]. Therefore, additional adjuvant chemotherapy might further improve the outcome in patients receiving definitive radiochemotherapy as well.

The results substantiate a considerable long-term recurrence-free-survival in patients treated with single-agent ifosfamide radiochemotherapy. Despite of the limitations of the retrospective comparison of different patient groups and the small case numbers, the data strongly suggest a better outcome of radiochemotherapy in combination with ifosfamide compared to radiotherapy alone and radiotherapy in combination with other radiosensitizers. The described treatment protocol should be tested in a greater patient population in order to generate more reliable data.

## Conflict of Interest Statement

The authors declare that they have no competing interests.

## Authors' contributions

All authors read and approved the final manuscript. FE: acquisition of data and data analysis, statistical analysis, writing and drafting of the manuscript. CM: acquisition of data and data analysis. ACM: data analysis, statistical analysis. MW: conception and design of the study. M JTH: conception and design of the study. CB: conception and design of the study. WB: conception and design of the study.
